# Phase manipulation of topologically engineered AB-type multi-block copolymers†

**DOI:** 10.1039/c9ra07734k

**Published:** 2019-12-18

**Authors:** Sai Li, Wei Tao, Ke Gao, Naveed Athir, Fanzhu Li, Yulong Chen, Jun Liu, Liqun Zhang, Mesfin Tsige

**Affiliations:** Key Laboratory of Beijing City on Preparation and Processing of Novel Polymer Materials, Beijing University of Chemical Technology People's Republic of China lj200321039@163.com; Beijing Engineering Research Center of Advanced Elastomers, Beijing University of Chemical Technology People's Republic of China; Engineering Research Center of Elastomer Materials on Energy Conservation and Resources, Beijing University of Chemical Technology People's Republic of China; Beijing Advanced Innovation Center for Soft Matter Science and Engineering, Beijing University of Chemical Technology 100029 Beijing People's Republic of China; State Key Laboratory of Organic-Inorganic Composites, Beijing University of Chemical Technology 100029 Beijing People's Republic of China; Department of Polymer Science, The University of Akron Akron Ohio 44325 USA; College of Materials Science and Engineering, Zhejiang University of Technology Hangzhou 310014 China

## Abstract

Recent advances in the fundamental understanding of the ordered phases of multi-block copolymers (MBCPs) at the molecular level have attracted considerable scientific interest in recent years. Herein, by employing molecular dynamics simulation, we focus on the four typical systems: linear alternating, branch-like, star-like AB-type MBCPs and linear copolymers filled with nanoparticles (NPs). First, we establish the phase diagram for the linear tetrablock copolymers (ABAB) as a function of the composition ratio between A- and B-block, exhibiting six typical phase states. Furthermore, increasing the mutual repulsive interaction strength, the temperature and the periodic dynamic shearing cycle result in the merging of spheres, presenting a clear beginning of the order-to-order transition (OOT) behavior. Second, we examine the branch-like and star-like copolymers and find that increasing branch density significantly leads to the occurrence of phase transition. Particularly, we illustrate that the sphere configurations of the MBCPs can be described in terms of tail, loop and bridge conformations. Increasing the number of distinct blocks in linear alternating copolymers results in an enhancement of the bridge conformation, in which case some spheres are separated to smaller ones. Furthermore, for the tail conformation, we present a unified theoretical framework to rationalize the topological state of the chain arrangements of spheres and infer that the entanglements within the internal reaction layer between different A-blocks result in the inhomogeneous distribution of the spheres sizes even with controlled molecular weight and composition ratio between each block. Finally, we find that the ABAB tetrablock copolymers filled with moderate spherical NPs exhibit a clear OOT from spheres to double gyroid or cylinders. We infer that the maximum amount of the B-block within the second and/or third layers for the filled spherical NPs connects different NPs effectively, leading to the complicated OOT behavior. Generally, this fundamental study could provide some guidelines for designing and fabricating high performance BCPs by manipulating the formation of the ordered phases.

## Abstract 2

Recent advances in the fundamental understanding of the ordered phases of multi-block copolymers (MBCPs) at the molecular level have attracted considerable scientific interest in recent years.

## Graphical abstract

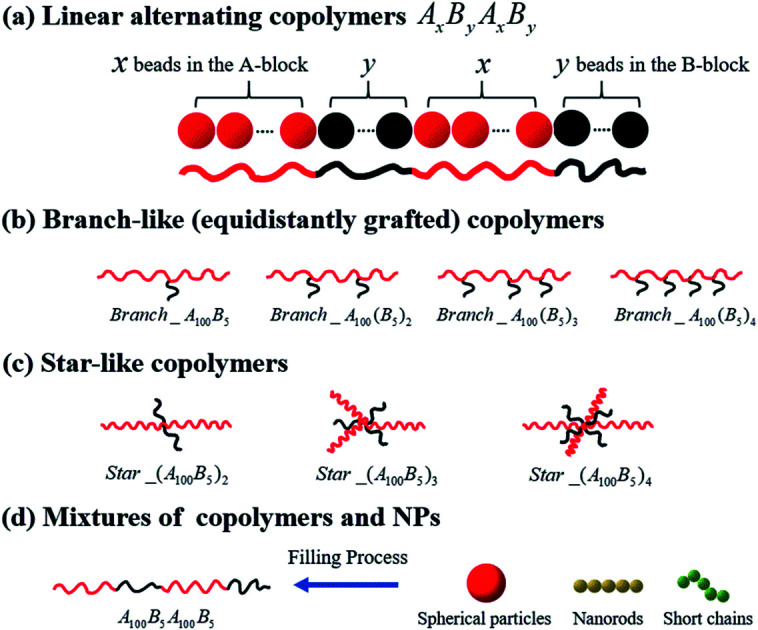

## 1. Introduction

Multi-block copolymers (MBCPs), consisting of polymer chains covalently linked as a series of two or more blocks, have gained a lot of traction in academia and industry due to the ability to precisely control over a variety of microscopic phase separation morphology, such as spheres, cylinders, double gyroid, lamellae and many other complex assemblies.^1–8^ Those highly stable morphology with no macroscopic phase separation make them a candidate for a wide range of industrial applications.^9–11^ However, due to the difficulty to synthesize complex block copolymers with precisely controlled architectures, comprehensive studies revealing the relationships between molecular architectures and morphology of MBCPs have been largely limited to a narrow range of diblock and triblock copolymers that have been well investigated in experimental and theoretical studies.^12–14^ Pioneers like Bates,^1^ reported an excellent review on the order-to-order transition (OOT) and order-to-disorder transition (ODT) behaviors of the simplest AB diblock copolymers in the strong^15^ and weak^16^ segregation limit, *i.e. χN* ≫ 10 and *χN* ∼ 10 respectively, where *χ* denotes the Flory interaction parameter and *N* is the degree of polymerization, according to the self-consistent field theory (SCFT).^17–19^ Notably, compared with the time-consuming experiments to address the effect of the chain architecture, SCFT has been remarkably used in predicting the equilibrium morphology and phase diagrams for diblock and triblock copolymers.^20^ Matsen^21^ expanded the current investigation and calculated the equilibrium phase diagram for a set of AB-type MBCPs by adopting SCFT, including the symmetric ABA, linear ABAB, 9-arm star-like, AB_2_ star-like and a comb architecture block copolymers. The results indicated that the phase diagram is relatively unaffected by the difference of the architecture for any AB-type MBCPs, but with different phase boundaries. However, more recently, Bin *et al.*^22^ had found novel hybrid morphology, such as lamellae–spheres phase, within a narrow stability region for ABAB using SCFT, which was also confirmed by Monte Carlo simulation.^23^ Though the results from these studies are very significant, the effect of block architecture on OOT behavior of MBCPs is far from fully understood. Furthermore, the phase diagrams calculated through SCFT have not fully explored the phase space and hence some potential phases may have been omitted.

On the other hand, with the emergence of new synthetic methods, it became possible for the preparation of AB-type MBCPs with more complex architectures, such as linear alternating, branch-like,^24^ star-like^25,26^ block copolymers, *etc.* Hermel *et al.*^27^ demonstrated a dramatic crossover from brittle to ductile behavior under excess strain as the linear alternating number increases for the linear alternating copolymers (LACs). Their small-angle X-ray scattering (SAXS) results indicated that the morphology of ABABA copolymers is dominated by a chevron structure with a variable angle to prevent brittle fracture when strained along the lamellae normal direction compared with the governing failure in the ABA triblock copolymers, and therefore ABABA presents great mechanical properties of elasticity and fracture toughness. Similarly, by blending triblock and multi-block copolymers with large linear alternating numbers, Tessie *et al.*^28^ investigated that the increasing linear alternating numbers of LACs could lead to the pinning of the interior blocks to two interfaces, which results in improvements in overall mechanical properties. These results proved that the architecture of MBCPs has a great influence on the micro domain alignment during flow or deformation and further affects their mechanical responses, although their precise relationship is not well understood. Moreover, Riess^29^ had reviewed the various synthetic methods of branch-like and star-like copolymers with different architectures which could self-assemble into diverse ordered microstructures. The increase in the number of possible morphology compared to their linear counterparts, MBCPs with various architectures result in complex phase behaviors. Bates *et al.*^30^ concluded that the molecular architectures of MBCPs depend on two parameters: (1) the number of chemically distinct blocks (*k*), and (2) linear *versus* branched sequencing of the blocks (*n*). Adding additional *k* or *n* to the MBCPs systems will significantly expand the possible molecular architectures and further influence its phase transition behavior.^31^

Indeed, developing a deep understanding of the OOT behavior of copolymers is quite complicated due to the fact that there are a number of molecular variables to consider, such as the degree of polymerization,^32^ introducing another type of covalently bonded blocks,^33,34^*etc.*

Furthermore, mixtures of copolymers and inorganic nanoparticles (NPs) have recently attracted more attention due to their wide and comprehensive applications.^35–37^ By coating gold NPs with either PS or PVP homopolymer and introducing them into poly(styrene-*b*-2 vinyl pyridine) (PS-PVP) diblock copolymers, Chiu *et al.*^38^ observed that those surface modified NPs can be localized within one or the other copolymer domain, as well as within the interface between the two blocks. Generally, the dispersion state of NPs depends strongly on the sizes,^39,40^ packing fractions^41^ and types^42–44^ of NPs, which is attributed to the system entropy and also essential to control the microstructure of the copolymer mixtures.^42–44^ Halevi *et al.*^45^ found that adding nanorods will have a bigger influence on the copolymer phase transition behavior, shifting the systems morphology to exhibit both lamellae and cylinders, compared with nanoparticle at the same filling fraction. The underlying reason could be filler geometry, meaning that nanorod fillers would introduce additional orientation entropy resulting from its anisotropy properties as compared with spherical NPs.^46^

Compared to experimental and theoretical work, computer simulations, especially Molecular Dynamics (MD) simulations, have gained unique advantages in revealing the connections between architectures and morphology due to their ability to precisely control polymer architecture, a factor that greatly influences experimental outcome. Keeping in mind the advantage of MD, the effect of architecture of the AB-type MBCPs alone, without changing other properties, on the phase diagram can be systematically investigated using MD.

In the present study, we aim to employ molecular dynamics simulation to first investigate the phase diagram of the LACs of the ABAB tetrablock copolymer type. Our focus is to explore the morphology state of different phases of ABAB, as well as their static and dynamic properties. We also provide a detailed discussion about the following three categories: (1) the conditions for the occurrence of OOT, such as the composition ratio of B- to A-block, the interaction strength between the blocks, temperature and the imposed periodic external-field; (2) the maximum critical island size to the sea-island structure; (3) and a mechanism to slow down the occurrence of the OOT. Secondly, we discuss the OOT occurrence of MBCPs for three different critical architectures, namely branch-like, star-like and linear alternating copolymers. Following, we determine three critical configurations to describe the possible chain arrangements in the morphology of spheres of MBCPs, such as tails, bridges and loops. We check one of the star-like copolymers by visualizing it, then modify the classic cone mechanism and display a suitable explanation of the spheres topological states, in order to address the issue that the sizes of spheres always disperse to some extent even with the controlled molecular weight and composition ratio of each block. Finally, we study the morphology transition behavior of mixtures of MBCPs and different types of NPs. The most interesting result is that we observe a transition trend from spheres to a complex morphology similar to the double gyroid or cylinders for the moderate spherical NPs filled system.

This work is organized as follows. In Sec. 2, we briefly describe the coarse-grained models of a series of MBCPs and its force-field parameters, such as the linear alternating, branch-like, star-like and mixtures of MBCPs with NPs. Results and discussion are given in Sec. 3. Followed Sec. 4 is Conclusion.

## 2. Simulation model and methods

### 2.1 Coarse-grained model

In this work, we present results using the coarse-grained (CG) MD simulations based on the Kremer–Grest bead-spring model of chains of multi-block copolymers (MBCPs). One polymer bead with a diameter of 1*σ* corresponds to 3–6 monomers in a realistic polymer chain such as polybutadiene, since one bead with a diameter of 1 nm roughly corresponds to 5 repeating units of polyethylene (with the carbon–carbon bond length being equal to 0.154 nm in polymer physics).^47^

We start by investigating the morphology transition behaviors of the linearly alternating tetrablock copolymers, such as A_*x*_B_*y*_A_*x*_B_*y*_. Note that in this work, the composition ratio of B- to A-blocks is always less than 1 (i.e. f* = *f*_B_/*f*_A_ < 1), meaning that A-blocks are always in continuous phase, while the B-blocks are in dispersed phase regardless of the variation in their concentration, unless otherwise noted. Secondly, we concentrate on a series of branch- and star-like MBCPs with different grafting densities to investigate the influence of architecture on the morphology state of copolymers. To avoid the influence of composition ratio, the A-block always consists of 100 Lennard-Jones (LJ) beads, while the B-block is composed of 5 LJ beads in this part. Moreover, for the branch-like copolymers, we only focus on the equidistantly grafting systems. To be more specific, for Branch_A_100_B_5_, we graft the B-block on the 50^th^ bead of the A-block, which almost guarantee the grafting point to halve the A-block equidistantly. In addition, the grafting points (GPs) are set to GPs = 33^rd^ & 66^th^ for Branch_A_100_(B_5_)_2_; GPs = 25^th^, 50^th^ & 75^th^ for Branch_A_100_(B_5_)_3_; GPs = 20^th^, 40^th^, 60^th^ & 80^th^ for Branch_A_100_(B_5_)_4_. As for the star-like MBCPs, the grafting points will set to be one of the two end beads of the A-block. In the last part, we consider the influence of polymer nanocomposites (PNCs) with different types of nanoparticles (NPs) introducing into the tetrablock copolymers A_100_B_5_A_100_B_5_, such as the spherical particles (SPs) with the radius *R*_*n*_ = 2*σ*; nanorods (NRs) and the short chains (SCs) which are both composed of 5 LJ beads.

**Fig. 1:**
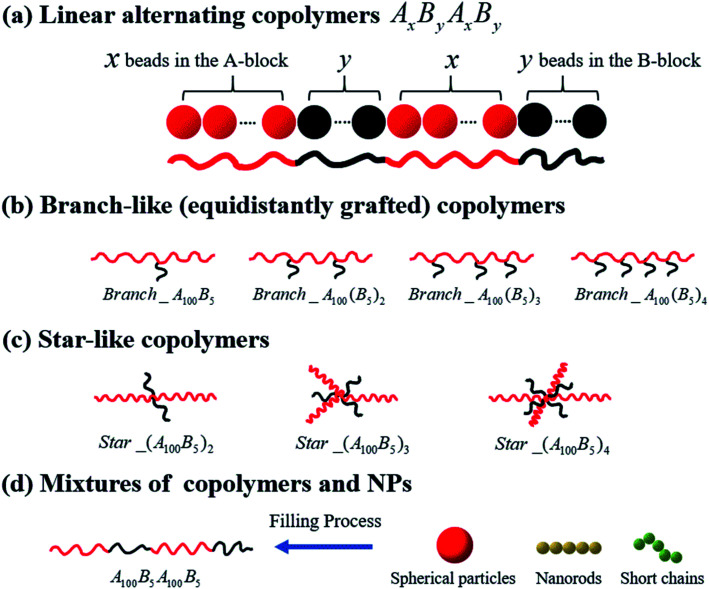
Schematics of the coarse-grained model of the copolymers studied in the present work, namely (a) linear alternating copolymers, (b) branch-like copolymers, (c) star-like copolymers, and (d) mixtures of copolymers and nanoparticles.

All systems discussed above are shown in Fig. 1, and the A- and B-block (represented by the red and black beads) are simplified as the red and black solid lines, respectively.

### 2.2 Simulation method

In all simulated systems, the diameter of copolymer beads is equal to *σ*, and the non-bonded interactions between them are modeled by the following truncated and shifted (TS) Lennard-Jones (LJ) potential:1
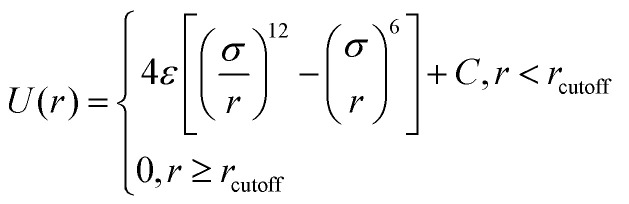
where *ε* is the energy scale of the pair interaction energy, and *σ* defines the length scale so that the simulated results are in reduced unit, *r* is the separation distance between two polymer beads, and the LJ interactions is cut off at different distances to model attractive or repulsive interaction. The repulsive interactions are simulated by setting 
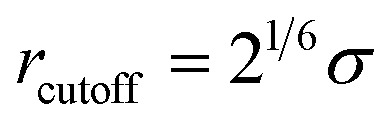
, whereas *r*_cutoff_ = 2.24*σ* and *r*_cutoff_ = 2.5*σ* represent a simulated short-ranged attraction and a long-ranged attraction separately as for the parameter of interaction strength *ε*, it should be noted that when mapping the bead-spring model to real polymers, the energy parameter *ε* is about 2.5–4.0 kJ mol^−1^ for different polymers. The constant *C* is added to guarantee that the potential energy is continuous at the cutoff distance. In the present simulation *σ* = *ε* = 1 was used for polymer beads. In order to model the different blocks of MBCPs, we introduce *r*_cutoff_ = 2.24 to mimic the attractive interaction between the two beads with the same type, such as A–A and B–B. On the other hand, *r*_cutoff_ = 1.12 is adopted to model the repulsive interaction between the two different types of beads, namely A–B and achieve microphase separation (see Table 1 in the ESI†).

**Table 1:** A description of copolymers with different architectures simulated in the current investigation

Branch-like copolymers	*N*_c-Branch_	Star-like copolymers	*N*_c-Star_	Linear alternating copolymers	*N*_c-LAC_
Branch_A_100_B_5_	600	Star_A_100_B_5_	600	Linear_A_100_B_5_	500
Branch_A_100_(B_5_)_2_	300	Star_(A_100_B_5_)_2_	300	Linear_(A_100_B_5_)_2_	250
Branch_A_100_(B_5_)_3_	200	Star_(A_100_B_5_)_3_	200	Linear_(A_100_B_5_)_3_	167
Branch_A_100_(B_5_)_4_	150	Star_(A_100_B_5_)_4_	150	Linear_(A_100_B_5_)_4_	125

Similarly, the filled spherical particles (SPs) are modeled as LJ hard spheres with the radius *R*_*n*_ = 2*σ*, therefore the mass of each SP is 64 times that of the polymer bead. Moreover, a modified LJ function that offsets the interaction range by *R*_EV_ is used to model the SP–polymer interaction and SP–SP interaction:2
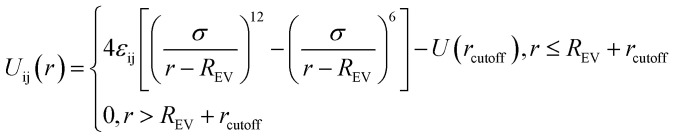


To consider the excluded volume effect of different interaction sites, the interaction range here is offset by *R*_EV_. For the SP–polymer interaction and SP–SP interaction, *R*_EV_ is set to *R*_*n*_ − *σ*/2 and 2*R*_*n*_ − *σ*, respectively. The actual cutoff is the sum of *r*_cutoff_ and *R*_EV_. The SP–SP interaction parameter and its cutoff distance are set to *ε*_nn_ = 1.0 and *r*_cutoff_ = 1.12*σ*, respectively. In addition, the interaction between SP and A-blocks are repulsive by setting *r*_cutoff_ = 1.12*σ*, while that of SP-blocks are attractive with *r*_cutoff_ = 2.24*σ*. Note that according to eqn (2) the real cutoff distance for SP–SP and SP-block interactions are 2*R*_*n*_ − *σ* + *r*_cutoff_ and *R*_*n*_ − *σ*/2 + *r*_cutoff_, respectively.

Meanwhile, the interactions between the adjacent bonded beads are maintained through the following harmonic potential:

3 *U*_bond_ = *K*(*r* − *r*_0_)^2^

where *r*_0_ is the equilibrium bond distance and *K* represents the spring constant of the bead-spring model. The parameters *r*_0_ = 1.0 and *K* = 200 were used, in which the spring constant is strong enough to prevent chain crossing. Note that the force-filed parameters of the short chains (SCs) are totally identical with that of the polymer matrix chains.

In order to model nanorods (NRs), we introduce the following harmonic potential to model their bending angle:
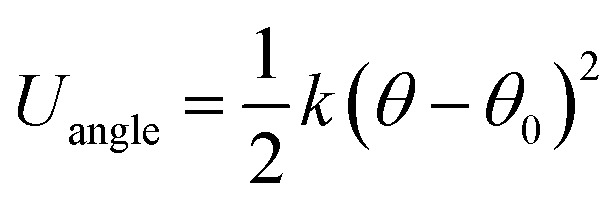
where *θ*_0_ is the equilibrium value of the angle which is set to be 180°, and *k* = 100 is used which makes sure that NRs can hardly bend at any time in this work. Since it is not our target to study a specific polymer, we use the reduced units, in which *ε*, *m* and *σ* are assumed to be unit (*ε* is the LJ energy parameter, *m* and *σ* are the mass and diameter of the monomer respectively). The means that all calculated quantities are dimensionless.

In our simulations, we adopted the isothermal–isobaric (NPT) statistical ensemble at the temperature *T* = 1.0 and *P* = 1.0 unless otherwise noted. The Nóse–Hoover thermostat and barostat are used to control the temperature and pressure. The velocity-Verlet algorithm is used to integrate the equations of motion, with a time step Δ*t* = 0.001. Periodic boundary conditions are enforced in current investigations of the simulation cell. In order to better observe the self-assembled phase state of each individual system, the equilibrium procedures are mainly composed of the following two steps. The detailed equilibrium process is in the ESI.† Note that for all discussion, such as the various property tests and the VMD presentation, we only focus on the second part of equilibrium procedures.

All the MD simulations were carried out by using the large scale atomic/molecular massively parallel simulator LAMMPS, which is developed by Sandia National Laboratories.^48^ More detailed descriptions of the simulation techniques in this work can be found in our previous studies.^49–53^

## 3. Results and discussions

### 3.1 Phase diagram of linear alternating ABAB tetrablock copolymers

**Fig. 2:**
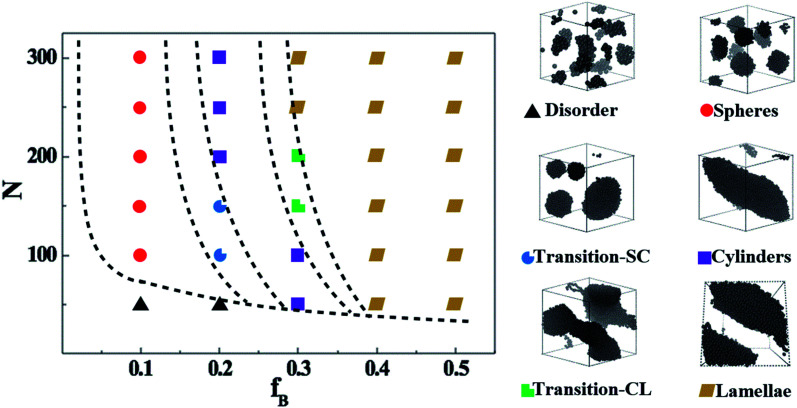
The phase diagram of ABAB tetrablock copolymers, depending on the composition ratio of the B- and A-blocks and the degree of polymerization (left); VMD of ordered phases of ABAB (right). Note: the B-beads are colored in black while the A-beads are transparent for better understanding.

We start by exploring the phase diagram of ABAB tetrablock copolymers based on the bead-spring model, as shown in Fig. 2. Since the phase diagram is left-right symmetry, we consider the composition ratio of B-blocks (*f*_B_ = *n*_B-beads_/(*n*_A-beads_ + *n*_B-beads_)) as the horizontal coordinate of the phase diagram, ranging from 0 to 0.5. The total number of beads along a copolymer chain (polymerization, *N*) is chosen as the vertical coordinate. The Visual Molecular Dynamics (VMD) snapshots shown on the right side of Fig. 2 clearly show that the ABAB copolymers could micro-phase separate into the following six morphology as a function of *f*_B_ and at a fixed *N*, including disorder (D), spheres (S), the transition order between S and C (transition-SC, T_1_), cylinders (C), transition order between C and L (transition-CL, T_2_), and lamellae (L). Among them, the morphology of the AB diblock copolymers has been extensively studied in the morphological transition behavior, namely D, S, C and L. For the morphology of L, our previous works have explained that when this metastable morphology of L undergoes a long period of periodic shearing or annealing, the energy barrier would be overcome and the interlayer connectivity would gradually disappear.^54^ As for T_1_, the island structure is roughly spherical, but the amount of islands is rather small compared with S and the size of each island varies greatly. On the other hand, T_2_ is the transitional order between C and L, where the separation trend of dispersed islands can be well observed. Note that although the fixed-size box is adopted in the current study, the enforced periodic boundary of the simulation cell makes sure there is no finite-size effect on the self-assembled morphology.

**Fig. 3:**
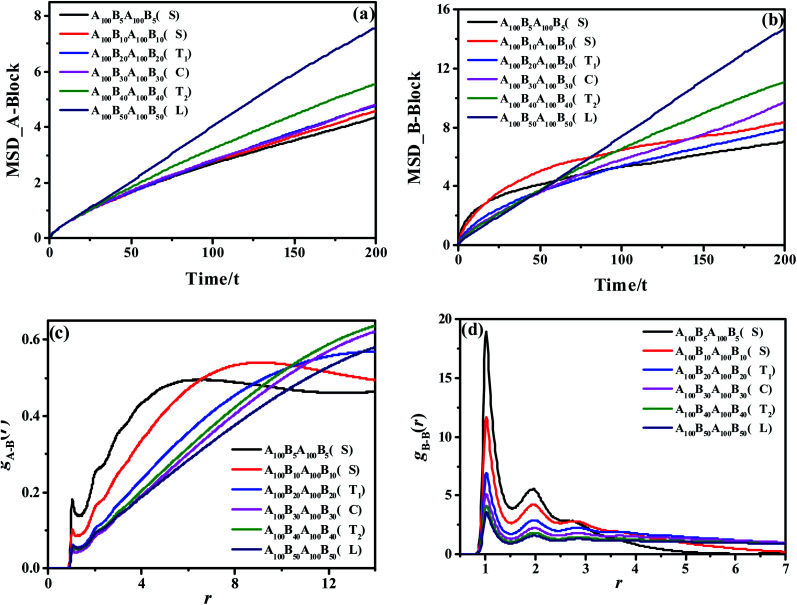
The MSDs of the A-block (a) and B-block (b) for A_100_B_5_A_100_B_5_, A_100_B_10*x*_A_100_B_10*x*_, copolymers, guaranteeing the OOT occurs. *τ* denotes the unit time; The RDFs of *g*_A–B_(*r*) (c) and *g*_B–B_(*r*) (d) for those copolymers.

Apart from the morphology of D, the transition of S → T_1_ → C → T_2_ → L is described as the order-to-order transition (OOT) in this work. To better understand the structural properties and motion abilities of the two types of blocks of various morphology under the occurrence of OOT, the mean-square displacement (MSD) and the radial distribution function (RDF) are calculated respectively, which are shown in Fig. 3. In this part, the following six copolymers are studied, namely A_100_B_5_A_100_B_5_, A_100_B_10*x*_A_100_B_10*x*_ (where *x* is an integer and is varied from 1 to 5); among which the morphology of A_100_B_5_A_100_B_5_ and A_100_B_10_A_100_B_10_ are both S. With increasing type-B beads (*i.e.* B-beads) *n*_B-beads_ at the fixed number of A-beads of *n*_A-beads_ = 100, the order of those systems guarantees the occurrence of OOT.

As for the mobility of different copolymers in line with various morphology, Fig. 3(a) and (b) display that the mobility of A-block increases significantly over time with the increase of the composition ratio of B-block, while that of B-block shows an interesting trend. From Fig. 3(b), both A_100_B_5_A_100_B_5_ and A_100_B_10_A_100_B_10_, which are referring to the morphology of S, present a sharp increase in the initial period of time (roughly *t* = 10*τ* for A_100_B_5_A_100_B_5_, and *t* = 20*τ* for A_100_B_10_A_100_B_10_), but witness a very slight increase in the long run. These results indicate that the morphology of S restricts the mobility of A-blocks but promotes the mobility of B-blocks in the beginning, and the mobility of A-blocks is highly dependent on the aggregated state of A-rich domains. On the other hand, the structural properties of A- and B-block are also characterized by calculating RDFs of *g*_A–B_(*r*) and *g*_B–B_(*r*) shown in Fig. 3(c) and (d), respectively, in which *g*_A–B_(*r*) refers to calculating the distribution probability of B-beads to the reference A-beads. In line with our prediction,^55^ the first peak in both RDF curves represents the closest distance for the monomers of a block chain, which is roughly the bond length *L*_bond_ = 0.96. For *g*_A–B_(*r*), the height of the first peak shows a decreasing trend with increasing the composition ratio of B-block, because of the decrease of the contact area between the two blocks as the OOT occurs. Interestingly, both A_100_B_5_A_100_B_5_ and A_100_B_10_A_100_B_10_ are decreased at the long calculated distance, mainly because of their special multipoint-aggregated dispersion state according to the morphology of S. In Fig. 3(d), there are several peaks existing in the *g*_B–B_(*r*), indicating several B-block layers around a reference B-bead. It is reasonable that the first few peaks of A_100_B_5_A_100_B_5_ and A_100_B_10_A_100_B_10_ are much higher than other copolymers, and the sharp decreasing trend of the second or third peaks also indicates the average size of spheres for A_100_B_5_A_100_B_5_ and A_100_B_10_A_100_B_10_. Note that the reason for why we consider the morphology of T_1_ as a special microscopic phase order is that it does not present a similar tendency of the RDF and MSD curves compared with that of S. However, it still remains unclear if both T_1_ and T_2_ are unique for the ABAB copolymers, but these two morphology are still good supplements to the classical phase diagram which deserve more attention in our future investigation.

Since the S morphology of ABAB presents unique properties both in the structural parameters and block segmental dynamics proved by MSD and RDF in Fig. 3, revealing the mechanism for the OOT taking place near the S morphology will be of great significance. Indeed, the key issue to track the OOT taking place near S is to precisely calculate the specific size of each spheres of the whole system, which is obviously a shortcoming in experiments. However, by adopting MD simulations, we are not only able to calculate the amount of B-beads in each sphere to characterize sphere sizes and plot the Normal Distribution (ND) curve similar to experiments, but also compute the Precise Distribution (PD) of the spheres from small to large. The horizontal coordinate of PD curve is chosen as the spheres ID in order from small to large, while the vertical coordinate is the amount of B-beads in each sphere. Note that PD curves can easily track any small changes of spheres sizes and amounts compared with ND curve.

**Fig. 4:**
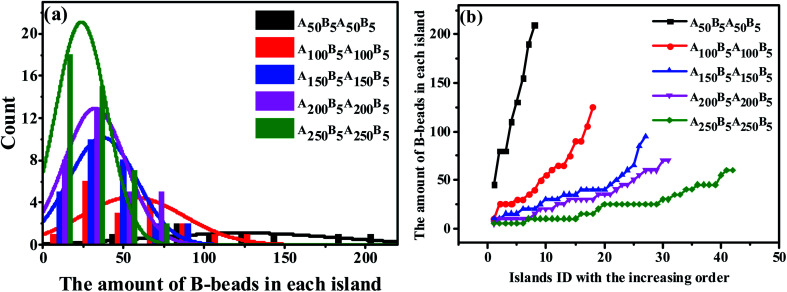
(a) The nominal distribution curve and (b) the precise distribution cure of the spheres sizes of A_50_B_5_A_50_B_5_, A_100_B_5_A_100_B_5_, A_150_B_5_A_150_B_5_, A_200_B_5_A_200_B_5_ and A_250_B_5_A_250_B_5_, ensuring the increase of the A-beads composition.

We start by investigating the effect of the composition ratio on the distribution state of spheres in the ABAB tetrablock copolymers. Fig. 4(a) indicates the ND curve of the spheres sizes as the OOT occurs. It is obvious that with the increasing number of A-beads at a fixed *n*_B–beads_ = 5 to ABAB, the distribution of peak points gradually becomes narrower and higher, meaning that the overall size of spheres becomes smaller and its distribution tends to be more homogeneous. As a comparison, we also plot PD curves of the same copolymers in Fig. 4(b), similar results could be found. Since the amount of B-beads in each system remains constant (which is also the precondition of plotting PD curves), the number of spheres formed by B-beads will increase as the size of the spheres become smaller. In addition, the peak location of each curve, which reflects the maximum critical island size (*i.e.* the size of the biggest sphere) to the sea-island structure, decreases shapely with decreasing *f*_B_. On the other hand, the average slope of PD curve can also reflect the polydispersity of the spheres sizes, meaning that PD curve with lower slope should result in a more uniform size for all spheres. Obviously, there is no absolute horizontal curve, indicating that with the controlled molecular weight of copolymers systems, the sizes of spheres are still dispersed to some extent, and the underlying reasons will be discussed in Sec. 3.3.

**Fig. 5:**
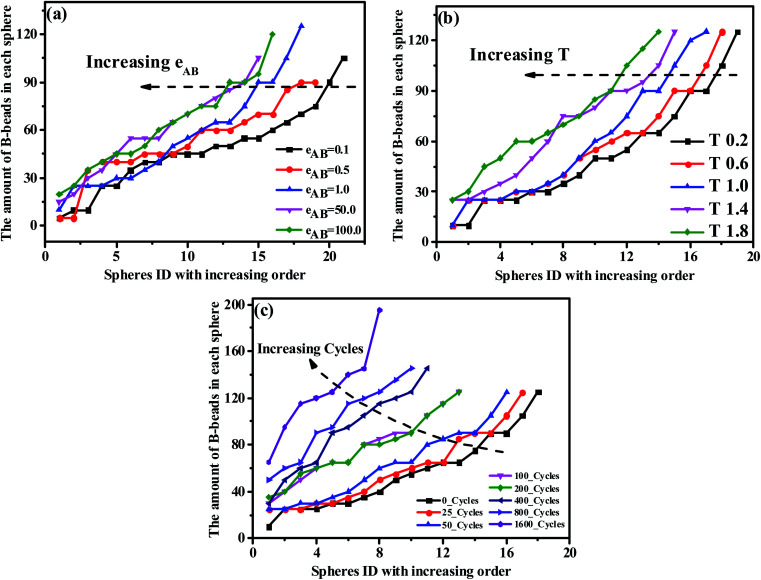
The precise distribution curves of A_100_B_5_A_100_B_5_ copolymers, with respect to (a) the repulsive mutual interaction, (b) temperature and (c) periodic shearing cycles.

From the perspective of microphase separation of the diblock copolymers, predicted by SCMF,^31,56,57^ the equilibrium-phase behavior depends on the following two aspects: (1) a lower interfacial energy depended on the minimizing contacts between the two unfavorable blocks (*i.e.* the A- and B-blocks), influenced by the Flory–Huggins parameter *χ*, (2) a higher configurational entropy depended on the stretching state of block chains, mediate by N* and f*. As a consequence, in order to check the influence of the unfavorable interaction on the morphology transition behavior, we display the PD curve of the tetrablock copolymers A_100_B_5_A_100_B_5_ as a function of *ε*_AB_, the energy scale of the LJ interaction between the A- and B-beads, in Fig. 5(a). It can be seen that increasing *ε*_AB_ leads to a left-ward shift for *ε*_AB_ ≤ 1, but then the shift is not significant. Furthermore, there is few significant change in the value of maximum critical island size, indicating the occurrence of fusion of one or two spheres. Note that when the mutual exclusion energy scale *ε*_AB_ is lower than 0.1 or it becomes attractive (*i.e.* we set the cutoff distance to *r*_cutoff_ = 2.5 form eqn (1)), the A_100_B_5_A_100_B_5_ copolymers will present a homogeneous and disordered distribution, which is not shown in this figure. On the other hand, we further investigate the influence of temperature as shown in Fig. 5(b), which illustrates a similar trend of the PD curves. Notably, the intersection between curves of *T* = 1.4 and *T* = 1.8 in Fig. 5(b) indicates that the new “fused” spheres will “dis-fuse” when the temperature continues to rise, due to its thermodynamic instability. Similarly, the periodic external-field effect, such as oscillatory shear deformation, is also considered in this work. Fig. 5(c) shows the PD curves of A_100_B_5_A_100_B_5_ under a series of periodic shearing cycles. It can be seen that the curves show a similar left-ward shift at the low shearing cycles (*i.e.*cycle < 100) compared with Fig. 5(a); when the cycles is above 200, the slope of the PD curve will increase rapidly along with the increase of the maximum critical island size, ensuring that the OOT occurs.

It is generally accepted that the micro-separation of MBCPs will gradually develop into various ordered morphology depending on a series of parameters that we have checked above, under the contribution of enthalpic and entropic among different blocks.^58^ Among those parameters, according to the strong-stretching theory introduced by Semenov,^59^ the interfacial tension between A- and B-blocks diminishes as the temperature increases, leading to the fusion of some spheres for the ABAB tetrablock copolymers. Similar trend could also be found with the increase of *f*_B_ or *ε*_AB_ and the imposed periodic external-field, resulting in a left-ward shift of the PD curve. Note that for the AB diblock copolymers, some literatures reported that the increased temperature will lead to the occurrence of the order-to-disorder transition (ODT).^60^ However, this trend has not been observed for ABAB in this work, partly because of their different configurations (see Sec. 3.3).

Importantly, we also explore the possibility to reduce the occurrence of OOT, and find that the left-ward shift of PD curves as a function of temperature and shearing cycles will reduce significantly, as expected, with 200 bonds crosslinking in A-rich domains to the A_100_B_5_A_100_B_5_ copolymers (shown in Fig. S1 of the ESI†). We believe that this observation is of good guiding significance for the application of MBCPs in a wide temperature range or at the fatigue state.

### 3.2 Effect of various molecular architectures on the OOT

In this section, we investigate the effect of a series of architectures on the morphology transition behavior of copolymers, namely the branch-like, star-like and linear alternating copolymers (LACs). Table 1 has a list of all the systems studied in this section with different molecular architectures, in which *N*_c_ represents the number of chains in each system. Note that by setting *N*_c_ of each system with different values, we can control the amount of type-B beads roughly of the same value, with the aim to plot the PD curve for each architecture. Taking an example of LACs, the amount of B monomers for the five LACs are shown as follows: 5 × 500 = 5 × 2 × 250 ≈ 5 × 3 × 169 = 5 × 4 × 125 = 5 × 5 × 100.

**Fig. 6:**
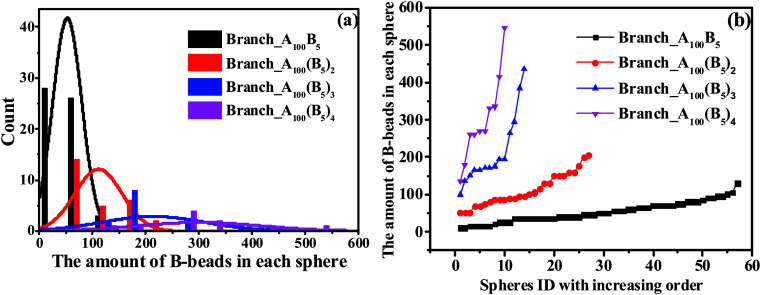
The nominal distribution curves (a) and precise distribution curves (b) for a series of branch-like copolymers.

**Fig. 7:**
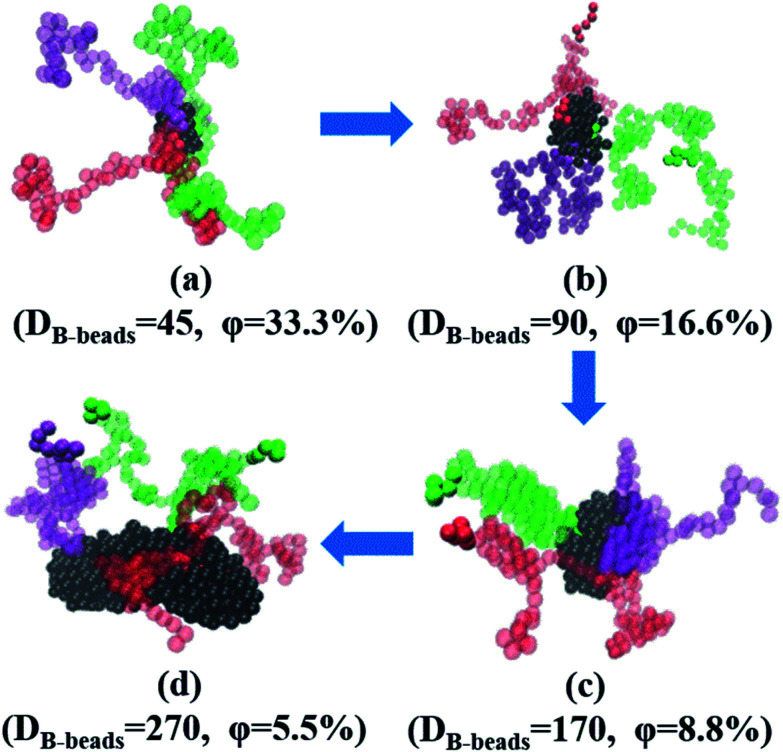
The flow chat of a series of snapshots of the four branch-like copolymers middle sized spheres/cylinders, (a) Branch_A_100_B_5_, (b) Branch_A_100_(B_5_)_2_, (c) Branch_A_100_(B_5_)_3_ and (d) Branch_A_100_(B_5_)_4_. Under each snapshots, *D*_B-beads_ denotes the number of B-beads, representing the size of each spheres/cylinders; while *φ* represents the ratio of three colored A-blocks to the total number of A-blocks linked with the spheres/cylinders, since we only present three colored chains to better observe the stretching state of A-blocks.

To begin with, we have checked the effect of the number of grafting B-blocks, and the ND and PD curves are shown in Fig. 6. Obviously, similar trends can be found in these figures compared with Fig. 4, and the maximum critical island size will undergo a sharp increase as the grafting number of B-block increases. The underlying reason is that with the increasing amount of grafting B-block, the composition ratio of B- to A-blocks is also increased sharply. Notably, with increasing the grafting number, the morphology of branch-like copolymers is transitioned from S to C. In order to directly track the occurrence of OOT, in Fig. 7, we further present the visualization of midsized spheres/cylinders for the four branch-like copolymers, where the middle spheres/cylinders are derived from the median of each PD curve shown in Fig. 6(b). The black beads in Fig. 7 represent the B-beads which form the spheres/cylinders for those systems; and the semitransparent red, green and purple beads represent the A-beads which links with the spheres/cylinders; while the solid red, green and purple beads indicate another spheres/cylinders which is covalently bonded with the colored A-blocks, proving the existence of configurations of bridges and loops with respect to Sec. 3.3. It can be seen that the size of spheres/cylinders and the amount of A-blocks to form the spheres/cylinders increase significantly as the grafting density increases.

Notably, we find that the location of grafting point will also greatly affect the microstructure of copolymers. Details about the non-equidistantly symmetric grafting investigation could be found in Fig. S2 of our ESI.†

**Fig. 8:**
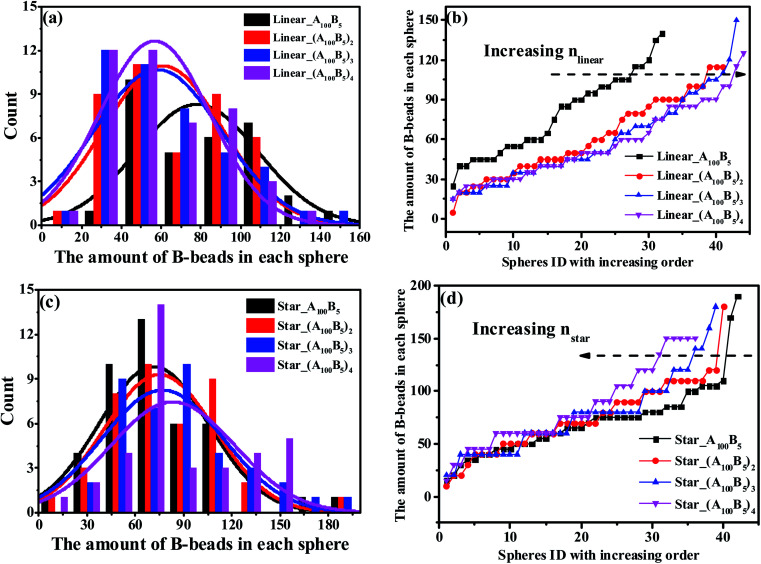
The nominal and precise distribution curves of a series of LACs with increasing *n*_linear_, as shown in (a) and (b); while (c) and (d) denote the star-like copolymers with increasing *n*_star_.

We further check the influence of LACs and star-like copolymers on the distribution state of spheres as shown in Fig. 8. Interestingly, no morphology transition behavior occurs with respect to the number of linear alternating copolymers (*n*_linear_) and the numbers of the star arms (*n*_star_), because of their constant composition ratio between the two blocks. In the meantime, focusing on the trend of each PD curve, it is found that Fig. 8(b) shows a right-ward shift as *n*_linear_ increases, while Fig. 8(d) presents a left-ward shift with the increase of *n*_star_. Moreover, from Fig. 8, with increasing *n*_linear_, the distance between the two adjoining PD curves decreases sharply, and the trend of the ND curves gradually becomes similar. The underlying reason might be due to the decreasing complexity of the molecular microstructures of spheres as *n*_linear_ and *n*_star_ increase (see Sec. 3.3). Note that for these two types of systems, the composition ratio of B- to A-blocks remains constant as f* = *f*_B_/*f*_A_ = 0.5/10, which might be due to the very slight changes of the maximum critical island size.

So far we have checked a series of factors that influence the maximum critical island size of the ABAB copolymers, as summarized in Table 2. Note that the primary factors would obviously have a big influence on the OOT occurrence of MBCPs.

**Table 2:** Variables influencing the maximum critical island size of MBCPs

Primary	Secondary
The composition ratio	The repulsive interaction, *ε*_AB_
The grafting density of B-blocks for the branch-like copolymers	Temperature, T
Periodical external-field with long cycles	The linear alternating number for LAC, *n*_linear_
	The star arms number for the star-like copolymers, *n*_star_

### 3.3 Developing a unified theoretical framework

**Fig. 9:**
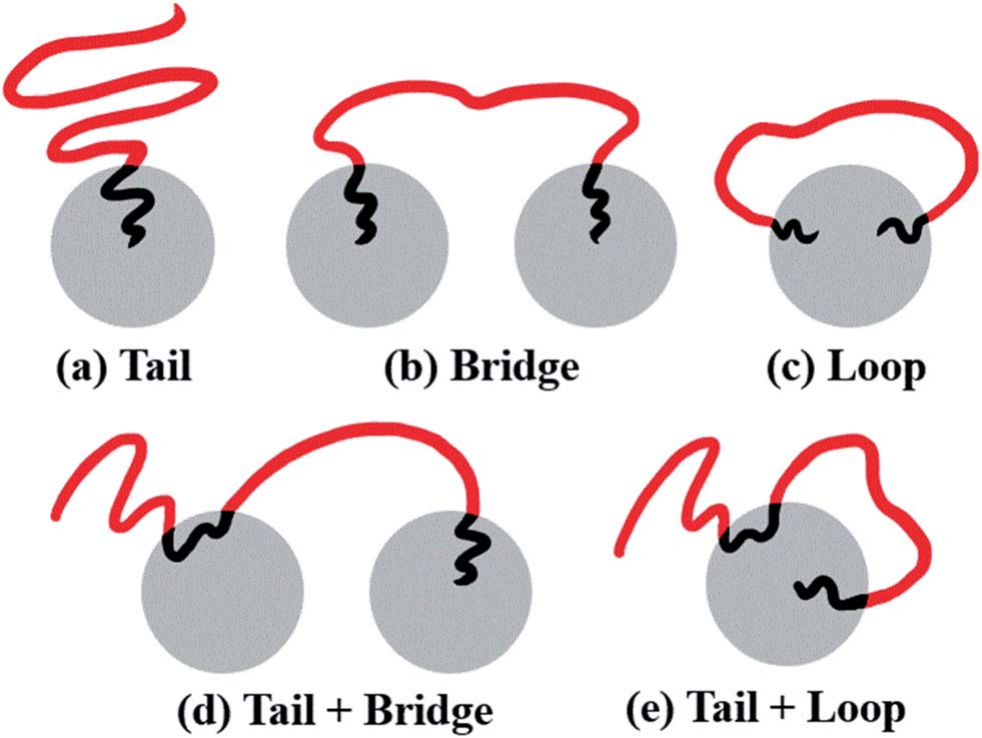
Schematic representation of a series of configurations, where (a) tail denotes the microstructure of AB diblock copolymers, (b) bridge and (c) loop denote BAB triblock copolymers, and (d) tail + bridge and (e) tail + loop represent ABAB tetrablock copolymers. The grey spheres denote the islands formed by B-blocks to each sea-islands structure. The red and black lines represent the A- and B-blocks, respectively.

From the molecular point of view, the configurations of different MBCPs are completely different. Taking the morphology of spheres as an example, the microstructure of the chain arrangements of A-blocks in AB diblock copolymers could be represented as tails (T), because there is only one end-bead of the A-block covalently bonded with B-block. As shown in Fig. 9(a), the B-rich domain forms the “islands” to the sea-islands structure, while the A-block is leaving within the “sea” structure, leading to an enhanced mobility of the A-block. By contrast, the mobility of the A-block in BAB triblock copolymers is restricted to some extent, due to its two end-beads linked with the islands. The A-blocks in which the two ends are pulled apart into the two different islands refers to bridge (B) shown in Fig. 9(b), while the two ends are anchored on the same island is represented by loop (L) shown in Fig. 9(c). Importantly, similar kinetics used to describe the dispersion state of polymer chains could also be found in some literatures.^61–63^

At a fundamental level, almost all microstructures of MBCPs can be described in terms of tail (T), bridge (B) and loop (L), if we only focus on the stretching state of A-blocks. For example, the configurations of the linear alternating ABAB tetrablock copolymers can be described as T + B and T + L, shown in Fig. 9(d) and (e). The underlying reason that is due to their simplest hybrid configurations of AB and BAB. Note that those different microstructures (T, B and L) can greatly influence the OOT behavior of copolymers, and further affect their viscoelasticity, mechanical strength, and other physical properties.^64^ For instance, the microstructure of bridge which effectively connects different spheres, could serve as a type of crosslinking chains and greatly improve the strain–stress behavior of copolymers.

In Fig. 8(b) and (d), we observe two different shift ward of PD curves for LACs and star-like copolymers as *n*_linear_ and *n*_star_ increase. From the viewpoint of microstructures, the right-ward shift of PD curve of LACs in Fig. 8(b) could be explained by the fact that the ratio of bridges increases with increasing *n*_linear_, which could divide some spheres, leading to an increasing amount of spheres. Moreover, we consider the reason for the significant difference between the PD curve of Linear_A_100_B_5_ and Linear_(A_100_B_5_)_2_ is the sudden introduction of the microstructure of bridges, leading to a remarkable change of its formed spheres amount. On the other hand, the configuration for the star-like copolymers should always be tail, and an increasing *n*_star_ only results in the reduction of A-blocks required for the spheres formation. This is the reason why Fig. 8(d) displays a left-ward shift and witness no unusual distance changes between any adjoining curves.

Indeed, there is a well-known cone mechanism indicating the copolymers chain arrangement in the morphology of spheres of the AB diblock copolymers,^60^ where the B-blocks trend to aggregate into spherical island when this AB diblock copolymers are highly asymmetric, representing a compromise among *χ*_AB_, f* and N*.^30^ However, it cannot address the issue raised in Sec. 3.1, *i.e.* the spheres sizes always disperse to some extent, which is also related to some experimental work,^65^ even with the controlled molecular weight and composition ratio of each block. Herein, we will present a unified theoretical framework complementary to the classical cone mechanism to explain the reason for the spheres of different sizes.

**Fig. 10:**
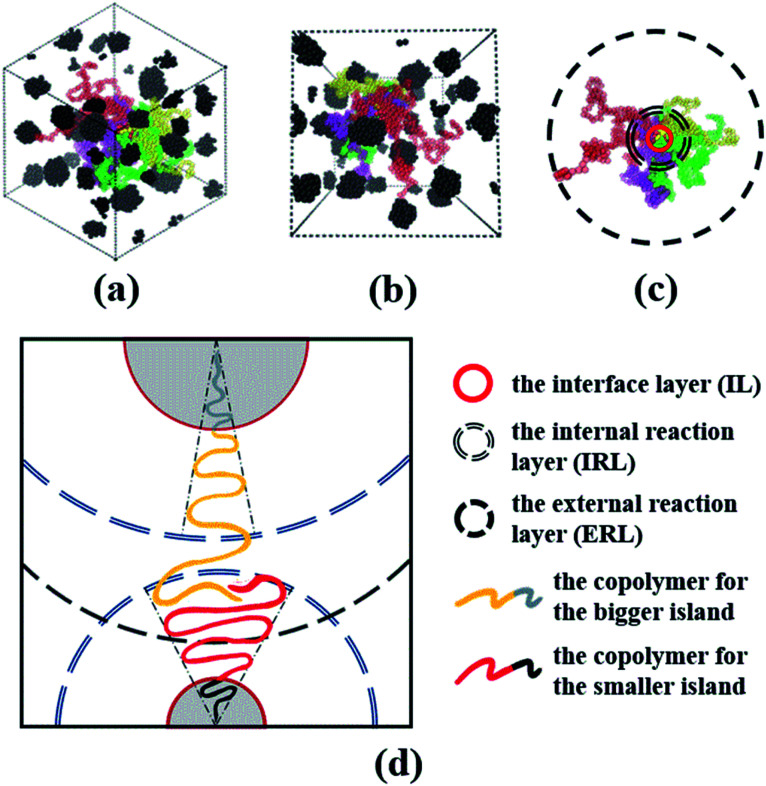
(a and b) Present the greatly dispersed state of A-blocks for only one spheres from different angles; (c) displays the stratification of three different layers, namely IL, IRL and ERL; (d) shows the mechanism of the modification cone model, where the A-block of the yellow-grey copolymers link with the bigger spheres and that of the red-black copolymers link with the smaller one. Note that composition ratio and polymerization of the two copolymers are identical, the difference of spheres sizes are attributed to the entanglements of the A-blocks within the IRL of the smaller sphere.

Now, we understand that this cone mechanism refers to the configuration of tail for A-blocks, and it can somehow explain the topological mechanism of the spheres of star-like copolymers. Therefore, we take Star_(A_100_B_5_)_4_ as an example to delineate the dispersion and stretching states of both A- and B-blocks for the spheres morphology, since only four copolymer chains form the middle sphere for this system. From the VMD figures of Fig. 10(a) and (b), it is clear that all spheres of Star_(A_100_B_5_)_4_ are well presented as the black aggregated beads, while the four A-blocks (*i.e.* the red, yellow, green and purple colored chains) which forms the middle sphere is displayed. It can be seen that those A-blocks is greatly dispersed throughout the matrix system, and therefore one can image the much higher level of tangled stretching states of all A-blocks which is covalently bonded with all those spheres. Due to A-blocks well dispersed state, following the idea of Gérard,^29^ we divide the cone model into the following three interaction layers as shown in Fig. 10(c): (1) the interface layer (IL), in which the two blocks are covalently bonded, (2) the internal reaction layer (IRL), where the entanglements of A-blocks linking with the core spheres mainly occurs within this layer, (3) the external reaction layer (ERL), where the entanglements of A-blocks linking with different spheres take place. We speculate that the ratio of the A-blocks within IRL to the B-blocks within IL, is the key to the formation of the core sphere, and only the two blocks within IRL and IL would follow the cone mechanism. Besides, the A-blocks between IRL and ERL would adhere to the model of random walk, interacting with other spheres' A-blocks due to the attractive interactions among them. Once those A-blocks are tangled with another spheres' A-blocks within IRL, the composition ratio of B- to A-blocks will be changed, leading to great changes in the angle and area of the cone model. That is the main reason for two aspects: (1) the different sizes of spheres even with the controlled molecular weight, (2) the irregular surface of some spheres. The main mechanism of this modified cone model is well displayed in Fig. 10(d).

### 3.4 Effect of the incorporation of the NPs on the OOT

**Fig. 11:**
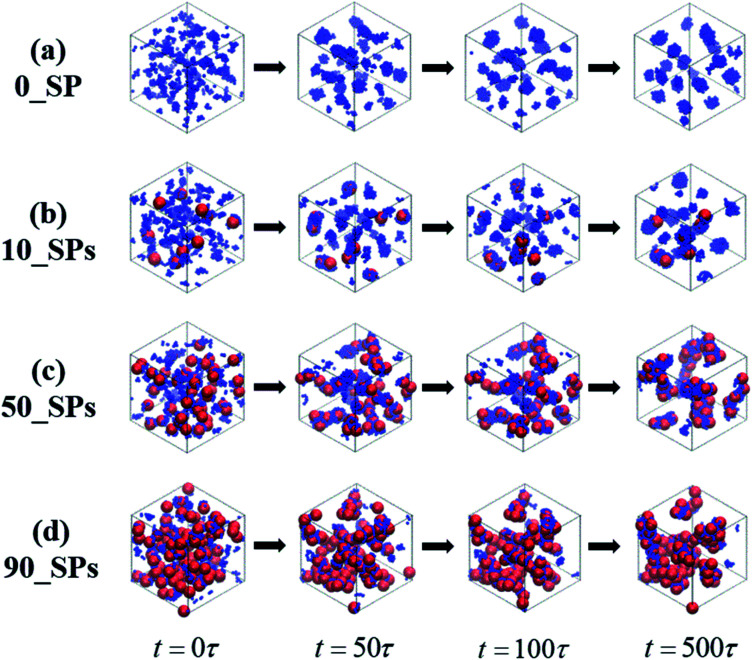
A series of flow chats of morphology transition behavior of the SPs filled mixtures over time, with different filing density, namely 0, 10, 50 and 100.

In this section, we introduce different types of NPs into the matrix composed of 100 chains of linear alternating copolymers A_100_B_5_A_100_B_5_, and try to explore the underlying reason for its ordered morphology. To begin with, Fig. 11 reports on the phase transition behavior of four different numbers of spherical particles (SPs) filled system over time. It can be seen that the microphase separation occurs in a very short time (*t* ≤ 50*τ*), with the introduction of the attractive interaction between B-beads and SPs and the repulsive interaction between A-beads and SPs. Specifically, different number of filled spherical particles would have a completely different effect on the phase transition of A_100_B_5_A_100_B_5_ as follows: (1) the flow chart of 0_SP represents the phase transition of pure copolymers of A_100_B_5_A_100_B_5_; (2) for 10_SPs (few filled system), the mixtures matrix also shows a morphology of S, where B-blocks are adsorbed on the surface of spherical particles; (3) for 50_SPs (moderate filled system), in which B-blocks are mainly adsorbed between two filled particles functioned as bridges, presenting a rough morphology between double gyroid and cylinders; (4) for 90_SPs (massive filled system), fillers tend to aggregate together on a large scale to present a disordered morphology.

**Fig. 12:**
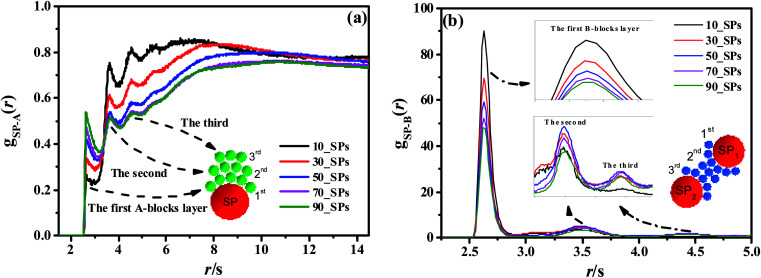
The RDFs of *g*_SP-A_(*r*) (a) and *g*_SP-B_(*r*) (b) for different SPs filling density. The red, green and blue spheres inside these figures denote the SPs, A-beads, and B-beads, respectively.

To reveal the underlying reason of the microphase transition behavior, we further calculate the RDF of *g*_SP-A_(*r*) as shown in Fig. 12(a). It can be found that several peaks exist in the short calculated distance, indicating different A-blocks layers to the middle reference SP. Among those layers, the first peak shows an increasing trend as the number of the filled SPs increases, while other peaks present a decreasing trend with increasing the filled density. The underlying reason is that B-blocks are competitively adsorbed onto the surface of the filled SPs as the first layer at the low filled density. Since the curve of the 50_SPs (moderate filled system) does not show a distinctive trend according to *g*_SP-A_(*r*), we further plot the RDF of *g*_SP-B_(*r*) as shown in Fig. 12(b). The height of the first shoulder peak decreases significantly with the filled density increasing, because the increasing amount of SPs relatively reduces the B-blocks coverage to the middle SPs. However, for the few filled system, the B-blocks only formed a first layer around the SPs, and the aggregated microstructure of SPs for the massive filled system also squeezes the B-blocks out of the collective structures, leading to the decrease of the height of the second and third peaks. Interestingly, the height of the second and third layers of the moderate filled system is ranked first as the effective layers. In order to explain its mechanism, we draw a simple schematic diagram in Fig. 12(b). Notice that the first, second and third B-blocks layers are referred to the SP_1_, and those layers would also be the third, second and first layers for the SP_2_. The effective second and third layers are functioned as bridges to connect the two SPs, especially for the second ones, leading to complex morphology similar to double gyroid or cylinders.

Finally, we also investigate the effect of different types of fillers to the same matrix of A_100_B_5_A_100_B_5_, such as the nanorods (NRs) and the short chains (SCs) (shown in Fig. S3 in the ESI†). In addition, the new formed spheres referred to the NRs/SCs filled mixtures would increase their mechanical properties, such as the stress–strain behavior, compared with the pure copolymers (shown in Fig. S4 in the ESI†). Due to space limitations, this part of work is well discussed in our ESI.†

## 4. Conclusions

Molecular Dynamics (MD) simulation is employed to investigate the ordered phases and its formation mechanism of MBCPs with different topologies and its nanocomposites. By presenting the phase diagram of ABAB, we find two novel ordered phases, such as transition-SC and transition-CL, in which the features of both phases are well evidenced by VMD snapshots. The MSDs and RDFs results indicate that studying the OOT taking place nearby the spheres phase has great significance in understanding the formation mechanism of each ordered phase. Therefore, we check the effect of the composition ratio of B- to A-block, the mutual interaction, the temperature and the imposed periodic external-field on the OOT behavior of ABAB. The simulation results suggest that the morphology and the critical island size of the aggregated B-blocks strongly depend on the composition ratio and the periodic external-field with long cycles. Furthermore, the imposed crosslinking bonds within the A-rich domains are confirmed to decrease to the occurrence of the OOT.

Secondly, the topological effect of MBCPs is also investigated by designing branch-like, star-like and linear alternating copolymers (LACs). We find that the increased amount of grafting B-blocks for the branch-like copolymers leads to the transition of OOT from spheres to cylinders due to the improved level of the composition ratio. On the other hand, we conclude that the configurations of chains arrangement of multi-block copolymers (MBCPs) can be described in terms of tail, loop and bridge conformations. As for the star-like copolymers and LACs, we find that the configuration differences among these copolymers further exhibit a contrary shift trend of their OOT behavior. The ascending linear alternating number leads to an increased ratio of the configuration of bridge, in which case some spheres are separated to smaller ones. But, no similar results were observed for the star-like copolymers, due to their constant microstructure of tail as the number of the star arms increases.

Particularly, we figured out a unified theoretical framework by modifying the classic cone mechanism to describe the formation mechanism of spheres. By dividing the cone model into three layers, such as the interface layer, the internal reaction layer and the external reaction layer, we point out that the entanglements within the internal reaction layer between different A-blocks lead to the inhomogeneous distribution of the spheres sizes even with the controlled molecular weight and composition ratio between each block.

Lastly, we probe the ordered phase transition behavior of copolymers filled with different types of NPs. For the moderate spherical NPs (50_SPs) filled system, we find a clear morphology transition from spheres to a complex morphology similar to the double gyroid or cylinders. However, no ordered phase transition behavior is observed for the nanorods or short chains filled systems.

## Conflicts of interest

There are no conflicts to declare.

## Supplementary Material

RA-009-C9RA07734K-s001
