# Supplementary material for: Phase manipulation of topologically engineered AB-type multi-block copolymers
Source: RSC Adv. 2019 Dec 18;9(72):42029–42. doi: 10.1039/c9ra07734k (PMC9076629; doi:10.1039/c9ra07734k)
Supplement: RA-009-C9RA07734K-s001 [file RA-009-C9RA07734K-s001.pdf]

Electronic Supplementary Material (ESI) for RSC Advances.
This journal is © The Royal Society of Chemistry 2019

Supplementary Material

Table of contents

1. The detail information of interaction potential energy between components

2. The detail information the units of Lennard-Jones units adopted in our simulation work

3. The equilibrium process of the systems

4.Plot of the precise distribution curve of the crosslinked $A_{100}B_5A_{100}B_5$ copolymers as a function of temperature and shearing cycles. (Fig. S1)

5.Effect of the non-equidistantly grafting system for the branch-like copolymers. (Fig. S2)

6.Effect of the different types of fillers on the morphology transition behavior of the $A_{100}B_5A_{100}B_5$ copolymers. (Fig. S3-S4)

1. The detail information of interaction potential energy between components

Table 1. Parameter of Interaction Potential Energy

A block-A block	A block-B block	B block-B block
LJ potential	LJ potential	LJ potential
ε = 1.0	ε = 1.0	ε = 1.0
r_{cutoff} = 2.24σ	$r_{cutoff(}$ = 1.12σ	r_{cutoff} = 2.5σ

2. The detail information the units of Lennard-Jones units adopted in our simulation work

We perform the coarse-grained MD simulation by using the simulation software package LAMMPS. In our simulations, the polymer chains were represented by the bead-spring model, in which the monomers were lumped together into spherical beads and the beads were connected through elastic springs. We used Lennard-Jones units (reduced units) for all units and Lennard-Jones (LJ) potential for all potentials, as mentioned in the manuscript. All quantities are reduced and unitless. Without the loss of generality, LAMMPS sets the fundamental quantities such as mass, sigma, epsilon, and the Boltzman constant equal to unity. The mass, distances, energies are multiple of these fundamental values. Although the reduced units are dimensionless, they have a corresponding relationship with the experimental units, as defined below:

length parameter: σ

mass parameter: m

well depth in LJ interaction: ε

reduced time: $t^* = t/\tau$; $\tau = \sigma\sqrt{m/\varepsilon}$

reduced temperature: $T^* = (k_B T/\varepsilon)$

reduced density: $\rho^* = \rho\sigma^3$

reduced frequency: $w^* = w\tau$

3. The equilibrium process of the systems

We describe the equilibrium procedures consisting of two steps. In the first step, our MD simulations are started from a non-overlapped configuration of all the polymer chains in a large simulation box. Three-dimensional periodic boundary conditions are implemented to eliminate the edge effect. By setting the cutoff distance between all beads equal to 1.12σ ($r_{cutoff} = 1.12\sigma$) and simulating the systems for a very long time of about 1.5×10^7 MD steps, the purpose of the process is to eliminate the influence of the initial structure. In the second step, we modify the LJ force-field parameters between different type beads. For example, we set the cutoff distance of the same kind of beads equal to 2.24σ or 2.5σ which represent a simulated short-ranged attraction and a long-ranged attraction, separately. Then, the simulation box is compressed under the *NPT* ensemble so as to increase the number density of polymer beads, keeping it around a reasonable value of $\rho^* = 0.85$. The velocity-Verlet algorithm is employed to integrate the equations of motion with a time step of $\Delta t = 0.001\tau$, and the temperature together with the pressure are

controlled via the Nose-Hoover thermostat and barostat. Each simulation system is equilibrated over a long time (5×10^6 time steps) to ensure that each polymer chain has moved at least $2R_{g_{RMS}}$ ($R_{g_{RMS}}$ is the root-mean-square radius of gyration). The corresponding total simulation time is in the order of ns.

4. Plot of the precise distribution curve of the crosslinked copolymers as a function of temperature and shearing cycles.

Fig. S1(a) and (b) plot the precise distribution (PD) curve of the same $A_{100}B_5A_{100}B_5$ copolymers systems with the effect of temperature and shearing cycles, after we crosslinked the A-blocks with 200 bonds. Due to the overlapping of some curves, we present the following method for better understanding. Taking an example of $T = 0.2/0.6$, it means the distribution state of $T = 0.2$ and $T = 0.6$ will share the same trend as shown in the red line in **Fig. S1(a)**. It is obvious that the crosslinking in seas structure (i.e. the A-blocks) can well reduce the possibility of the fusion of some islands, and therefore the maximum critical island size will remain constant even at a very high shearing cycles.

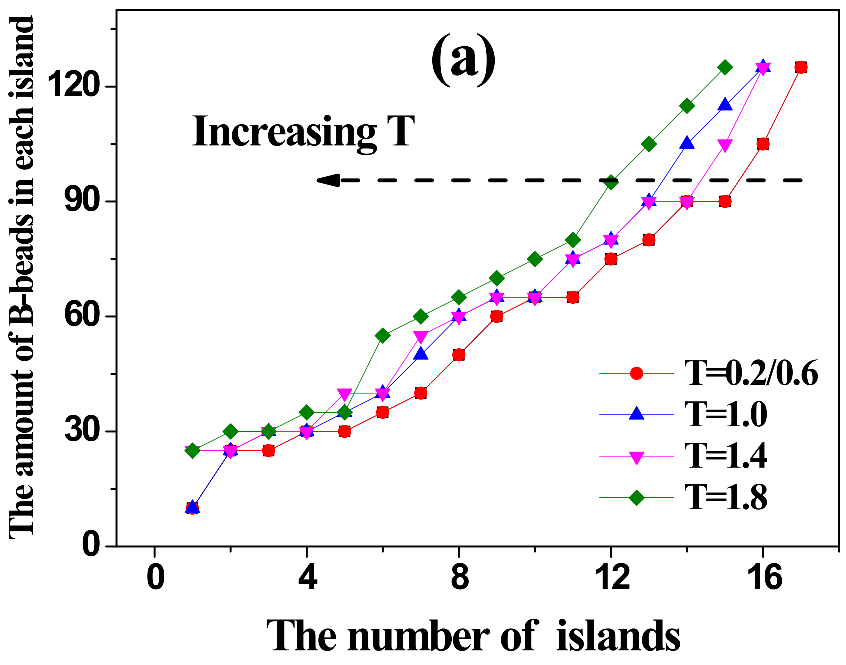

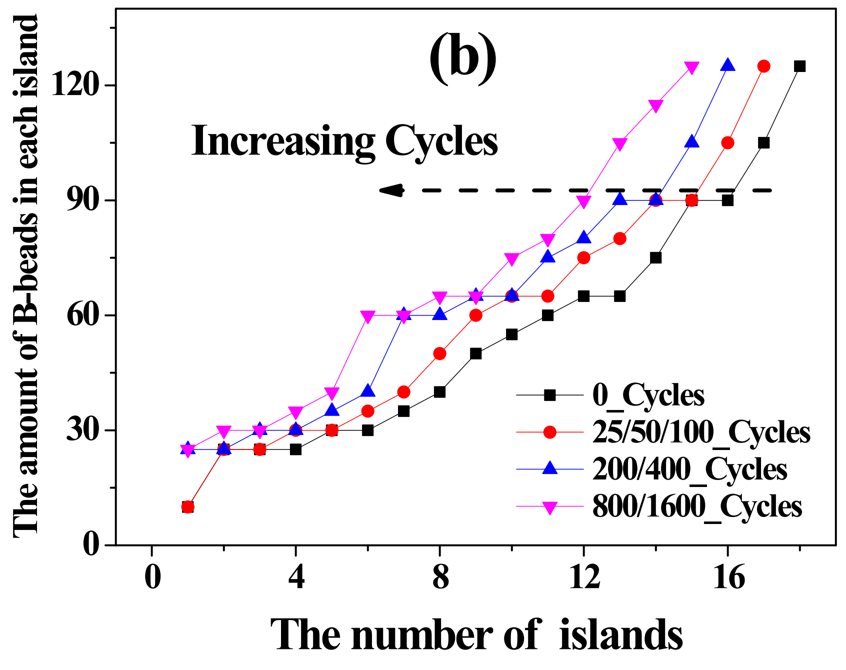

Fig. S1 The precise distribution curves of the crosslinked $A_{100}B_5A_{100}B_5$ as a function of Temperature (a) and shearing cycles (b).

5. Effect of the non-equidistantly grafting system for the branch-like

copolymers.

For the branch-like copolymer $Branch\_A_{100}(B_5)_2$, there are only two configurations from the molecular insight, namely T+B+T and T+L+T shown as **Fig. S2(a) and (b)**. Furthermore, **Fig. S2(c)** presents a series of ratio of these two types of microstructures with different symmetric grafting points, where the symmetric grafting method means that if the first grafting point is the 33rd bead of the A-block, the other grafting point should be 67th bead. Note that $Branch\_A_{100}(B_5)_2$ would be the linear BAB triblock copolymer if the first grafting point is 1st bead of the A-block. It can be seen that $Branch\_A_{100}(B_5)_2$ with the 15th grating beads is of the biggest ratio of the microstructure of Bridges, meaning that it would have the greatest stress-strain behavior, due to the fact that the bridges between each island will be treated as the crosslinks when it undergoes a uniaxial tension test.

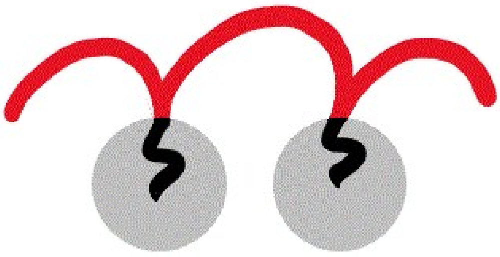

(a) Tail + Bridge + Tail

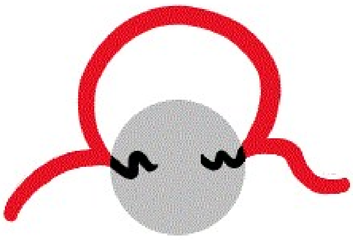

(b) Tail + Loop + Tail

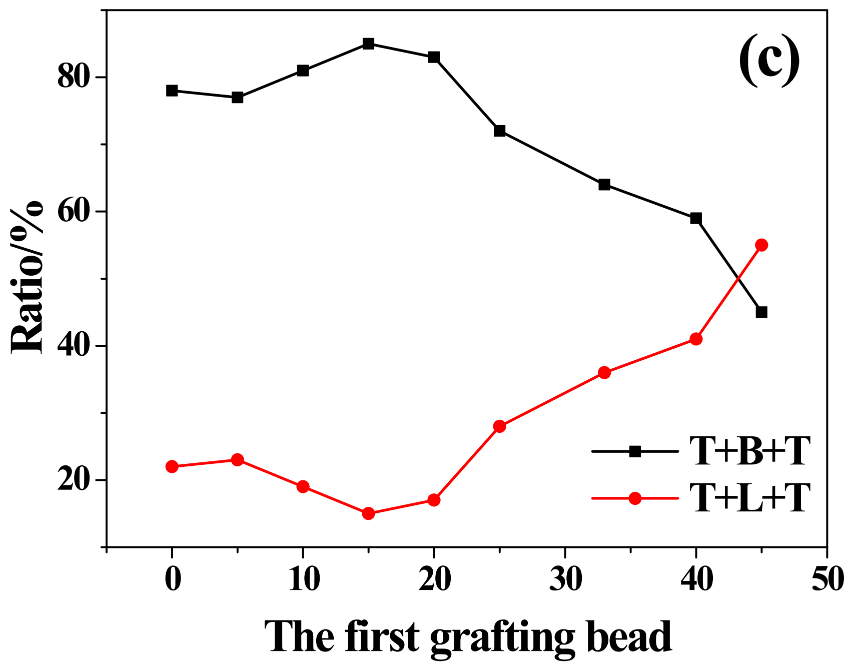

Fig. S2 (a) and (b) present the two different configurations for the branch-like copolymers $Branch\_A_{100}(B_5)_2$, (c) Plot of the different ratios of the two microstructures with different first grafting bead to $Branch\_A_{100}(B_5)_2$.

6. Effect of the different types of fillers on the morphology transition behavior of the $A_{100}B_5A_{100}B_5$ copolymers.

In this part, we first investigate the influence of the Nanorods (NRs) and the short chains (SCs) filled mixtures on their morphology transition behavior at the 50 and 100 filling density, as shown in **Fig. S3**. However, the result indicates that the filled NRs and SCs can neither change the microstructure of $A_{100}B_5A_{100}B_5$, namely S. It can be seen that the filled NPs and SCs both closely entangle with B-blocks to form the island structure. Additionally, we suspect that this new formed island could improve the

stress-strain behavior of the matrix, which is shown in **Fig. S4(a)** as expected. Notice that the rigidity of the filler is further enhanced the mechanical properties of the system as shown in figure. In order to reveal its reinforcing mechanism, we further plot the contribution degree of the island versus elongation ratio of the five filled systems, as shown in **Fig. S4(b)**, to check the contribution of fillers to the stress-strain behavior. It is obviously checked that the contribution ratios of NRs or SCs filled systems are all higher than that of the pure $A_{100}B_5A_{100}B_5$ copolymers in the uniaxial tension test over the elongation ratio. Note that all the five curves in **Fig. S4(b)** explain a decreasing trend with increasing elongation. It is revealed that the island structure would plays an important role at the beginning on a tension test. Because of this the copolymer network is not fully stretched, whereas the continuous phase (i.e. the A-blocks) is dominant to the stress-strain behavior as the strain increases.

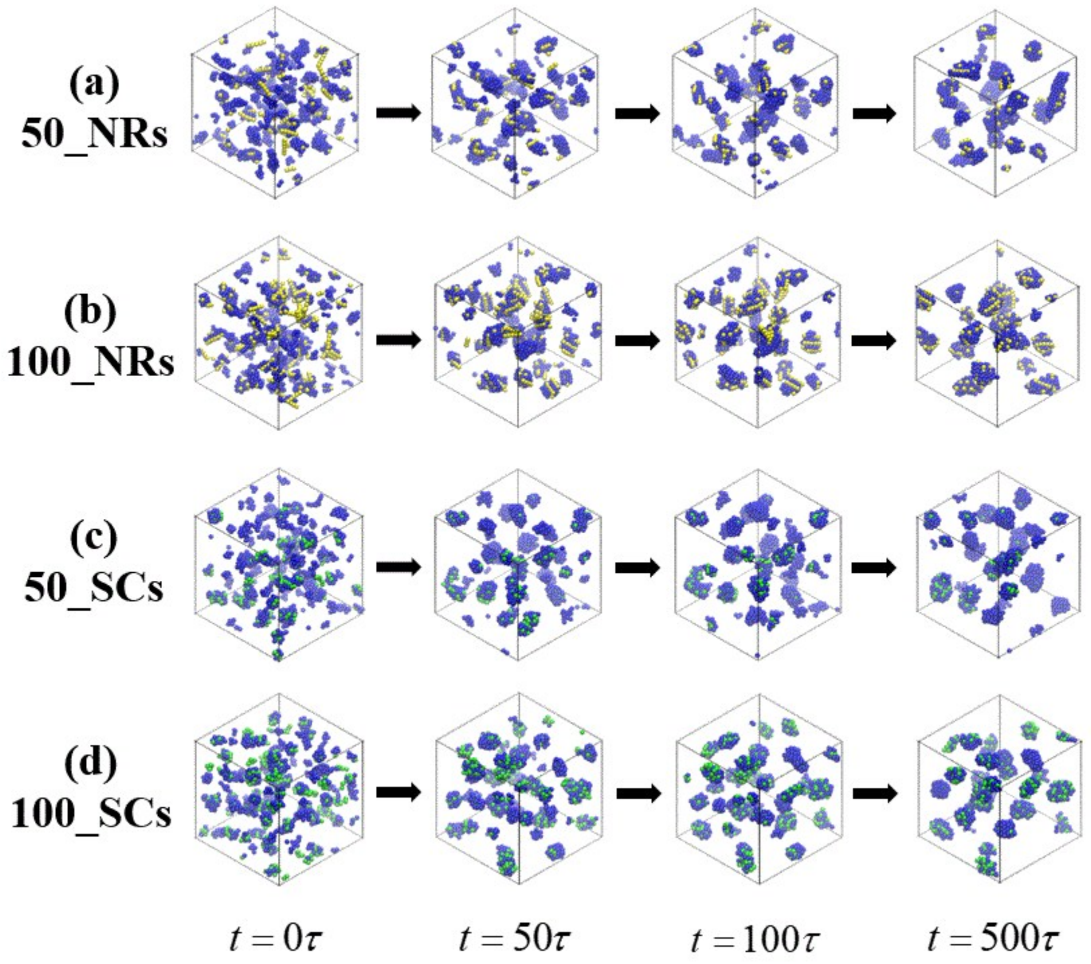

Fig. S3 A series of flow charts of morphological transition behavior of four different filled systems, namely (a) 50_NRs, (b) 100_NRs, (c) 50_SCs and (d) 100 SCs.

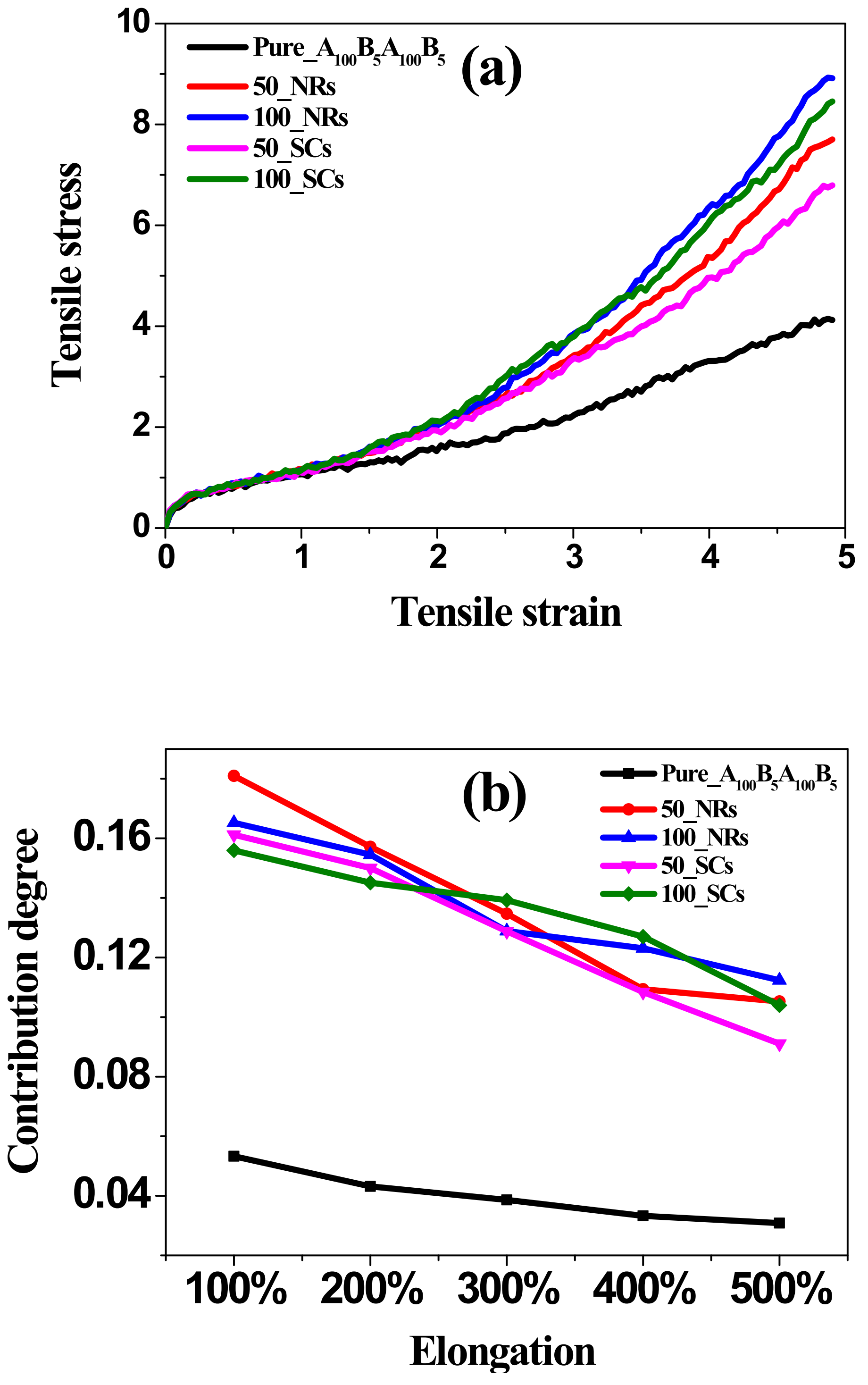

Fig. S4(a) The stress-strain behavior of a series of copolymers, namely pure $A_{100}B_5A_{100}B_5$ copolymers, 50_NRs, 100_NRs, 50_SCs and 100_SCs different filling density systems; (b) The contribution degree of the five copolymers to the stress-strain behavior at a series of elongation ratios.
